# New Politics, an Opportunity for Maternal Health Advancement in Eastern Myanmar: An Integrative Review

**Published:** 2014-09

**Authors:** Adam B. Loyer, Mohammed Ali, Diana Loyer

**Affiliations:** Curtin University of Technology, Australia

**Keywords:** Government, Human rights violations, International aid, Liberalization, Maternal health, Maternal mortality, MMR, Policy, Pregnancy, Burma/Myanmar

## Abstract

Myanmar (formerly Burma) is a southeast Asian country, with a long history of military dictatorship, human rights violations, and poor health indicators. The health situation is particularly dire among pregnant women in the ethnic minorities of the eastern provinces (Kachin, Shan, Mon, Karen and Karenni regions). This integrative review investigates the current status of maternal mortality in eastern Myanmar in the context of armed conflict between various separatist groups and the military regime. The review examines the underlying factors contributing to high maternal mortality in eastern Myanmar and assesses gaps in the existing research, suggesting areas for further research and policy response. Uncovered were a number of underlying factors uniquely contributing to maternal mortality in eastern Myanmar. These could be grouped into the following analytical themes: ongoing conflict, health system deficits, and political and socioeconomic influences. Abortion was interestingly not identified as an important contributor to maternal mortality. Recent political liberalization may provide space to act upon identified roles and opportunities for the Myanmar Government, the international community, and non-governmental organizations (NGOs) in a manner that positively impacts on maternal healthcare in the eastern regions of Myanmar. This review makes a number of recommendations to this effect.

## INTRODUCTION

Myanmar (formerly Burma) is a populous and impoverished southeast Asian country, with a long history of military rule and political conflicts characterized by widespread human rights violations. Myanmar's population of pregnant women are particularly at risk, and that risk is significantly higher among pregnant women of ethnic minority groups in the eastern provinces. Very limited data for the strife-ridden eastern provinces have made it difficult to establish a pattern of the prevalence of maternal mortality in Myanmar. Officially, the country's Central Statistical Organization reported the nation's urban maternal mortality ratio (MMR) to be 123 per 100,000 livebirths and the rural MMR to be 157 (1). International bodies, such as the WHO, UNICEF, UNFPA, and World Bank, however, suggest that the overall MMR may be as high as 200 (2,3). The Back Pack Health Worker Team (BPHWT), one of the few humanitarian assistance organizations that operate in the eastern regions of Myanmar, has collected data suggesting that the MMR in the eastern provinces may be as high as 721 per 100,000 livebirths (4). This integrative review seeks to examine the factors contributing to the higher MMR in eastern Myanmar (Kachin, Shan, Mon, Karen and Karenni regions).

The major medical causes for maternal deaths in Myanmar are listed as: 31% postpartum haemorrhage (PPH), 17% hypertension-related disorders, and 10% sepsis relating to abortion (5). However, for the eastern regions, a more accurate picture, as reported by the BPHWT (2011), may be: 23% PPH, 38% obstructed labour, 23% eclampsia, and 8% fever.

With an estimated per-capita GDP of US$ 742 (6), approximately 25% of Myanmar's people live below the poverty line (7). In the eastern regions, poverty is compounded by the food insecurity often brought on by conflict (8). Public healthcare facilities are understaffed and underresourced and, while nominally free, the reality is that many health services require a user fee (9) that the Government has imposed as a so-called community cost-sharing scheme (10). Family planning services, such as contraception, are difficult to obtain, particularly in the eastern regions (8). As recently as 2000, family planning services were virtually unavailable in Myanmar. According to the Ministry of Health, the national unmet need for family planning is 17.7% (11), a rate similar to that found by the UN (12). However, the unmet need in the eastern regions has been documented at greater than 60% (13).

Many women in Myanmar resort to illegal abortions as the primary means of contraception (14). Officially, Myanmar makes antenatal care available to 83% of pregnant women nationally (5); however, other estimates from the UN suggest that the national rate may be closer to 76% (12). In the eastern regions, Mullany and colleagues found that, prior to any intervention on their part, antenatal care in that area covered less than 40% of pregnant women (13). Even with antenatal care coverage, many mothers still suffer from poor nutrition, anaemia, or malaria. A significant lack of skilled birth attendants has been noted; one midwife is often responsible for five to 16 villages (15).

The Ministry of Health, quoting data from 2008, suggested that 76% of pregnant women received skilled attendance (11). Other non-governmental bodies suggested that it may be closer to 57% (12,16). Grundy *et al.*, quoting a 2011 WHO report, suggested that as many as 73% of Myanmar's women gave birth in village homes in the care of non-professionals (17). In contrast, skilled attendance in the eastern regions has been documented as low as 5% (13). Emergency obstetric care is inadequate nationally (5).

Accessing care is particularly difficult in the eastern provinces since the few available hospitals are at great distances from villages (18) and are dangerous to access due to landmines and ongoing conflict between the military and ethnic militias, seeking greater autonomy or independence for their regions. Ethnicity further places women at risk since the Myanmar Government consistently appears to provide fewer services to ethnic minorities. Healthcare is more deficient in regions of ethnic minority groups (19), and infrastructure is particularly poor in the rural areas of these regions (20).

Myanmar has struggled under military rule for more than 50 years. The military consumes 40% of the national budget while merely 2.2% is allocated to healthcare (5). The long-standing military government in Myanmar was also well-known for human rights abuses revolving around ongoing conflict with ethnic minorities (13). Research by the BPHWT has linked poor health indicators to the human rights abuses experienced in the eastern provinces (4,8). It is in this context that recent political liberalization in Myanmar has been encouraging. In March 2011, a nominal civilian government, headed by President Thein Sein, was elected, representing Myanmar's first non-military rule since 1962. The new government has sought to demonstrate leniency, recently releasing over 2100 political detainees (21). Among those released from house arrest was Aung San Suu Kyi, an internationally-renowned democratic figurehead. Finally, in recent years, the Myanmar Government has signed ceasefire treaties with several ethnic rebel groups (22).

The principal aim of this paper is to carry out an integrative review of the literature on the current status of maternal mortality in eastern Myanmar in the context of armed conflict between various separatist groups and the military regime. In the process, underlying factors contributing to maternal mortality in eastern Myanmar are examined. The authors discuss possible opportunities that liberalization may make available to the Myanmar Government, the international community, and non-governmental organizations (NGOs) in the fight to reduce maternal mortality.

## MATERIALS AND METHODS

### Literature search

This integrative review of literature sought qualitative, quantitative and unpublished studies, and grey literature (grey literature refers to documents of the Ministry of Health, organizational reports, country documents, book chapters, and newspaper articles). An exhaustive electronic search, in English, through scholarly databases (Medline and Embase) and full-text journal databases (Proquest, Ovid and Science Direct) was conducted using the following key words: Burma, Myanmar, childbirth, civil war, death, development agencies, international aid, government, liberalization, maternal deaths, maternal health, maternal mortality, MMR, political unrest, policy, and pregnancy. The corresponding authors of publications in the field of maternal mortality in Myanmar were contacted via a standardized email for any outstanding unpublished articles. Of those authors who responded, only one had authored additional material on the subject. Grey literature was examined with Google and Google Scholar search engines, using the same search terms mentioned above. Unpublished documents were sought in electronic theses libraries. Hand-searching of relevant journals was also completed for the period spanning the six months prior to 31 July 2012 to ensure that any recent publications not yet available in databases were not overlooked. No relevant documents were found. All publications that entered the second phase of screening (see below) were examined for relevant titles from their references lists.

**Table 1.** Data-collection tool

Author .........................	
Year ............................	
Title ...........................	
Type of study: (report, quantitative, survey, qualitative…)	
Direct medical causes	1b^1^. Postpartum haemorrhage
	1c. Sepsis
Access to MHC services	1t. Poor transportation
	1w. Cost for use of healthcare 1-hinders usage
Availability and use of MHC services (incomplete abortion)	1d. Ministry of Health facilities 1-fewer facilities in E 2-no/lack of facilities 3-poorly functioning
	1f. Poorer access to health services
	1x. Few financial resources for health
	1y. Illegal, unregulated medicines
	2a. Abortion 1-anti-abortion policy-dangerous abortion
	2b. Natal policy (allows for birth spacing)
	2c. Sexual health services and contraception
	2d. Ministry of Health facilities 1-fewer facilities in E 2-no/lack of facilities
	1h. HR shortage
MHC services	1a. Dangerous abortions
	1e. Location of birth 1-home most common 2-jungle
	2f. Birth attendants
	1l. Skilled attendance at birth (any level)
	1m. Antenatal visits (any number)
	1.i. Lack of EOC services
	1s. Postnatal care
Health of mother (pre-existing)	1o. Iron supplements
	1q. Anaemia
	1j. High anaemia
	1n. Malaria presence/malarial insecticide-treated bednets
	1k. High malaria
	1r. Age at marriage
Contraception	1p. Unmet contraceptive need
	1u. Modern contraceptive usage
Lack of data collection	2g. Statistics 1-E not in census 2-lack of reliable data
NGO limitations and hindrances	2e. Geographic distribution of NGOs
	1g. Difficult for NGOs to access
	2h. Humanitarian space 1-not given access
	2i. NGOs not free to work
	2j. Military autonomy/self-reliance
	2k. Cumbersome procedures for NGOs
	2l. Visa restrictions to NGO staff
Sexual violence	3b. Rape 1-2-sexual violence
	4a. Sex slaves
	3s.i. Sexualized torture
	4b. Human trafficking
Relocation/ displacement	3d. Relocation 1-forced
Personal violation/violence	3f. Violence 1-to civilians
	3p. Human minesweepers
	3i. Torture 1
	3j. Killing 1
	3u. Intentional targeting of civilians
Targeting of medical staff, buildings, supplies	3s^2^. ii. Restricted movement of medical personnel
	3h. Detain, kill medical workers 1-happens
	3g. Destroy medical supplies 1-destruction
Conflict-related barriers to accessing MHC	3c. Clinic accessibility 1-landmines 2-patrols
	3s.iii. Restricted movement 1-civilians
	3m. Detain 1-civilians
	3k. Landmines 1-hurt civilians 2-hurt med workers
	3l. Ongoing conflict
Removal of food and money sources	3a. Food security 1-burning of fields 2-stealing of crops 3-destruction crops
	3e. Forced labour 1-occurs 2-porter service 3-road building
	3n. Conscription
	3o. Burning/destruction of homes or villages
	3q. Human shields
	3r. Theft 1-fines 2-property 3-taxes

^1^1a is not missing, it is the first item in the ‘MHC Services’ section of the table. The items are not in sequence because, when these were grouped into broader themes (as described in the methods section), these maintained their numbering and lettering

^2^Restricted movement of medical personnel—travel limitations for medical personnel due to conflict, threats of violence, landmines, etc.

### Screening of the studies

Documents selected for the review included published journal articles (n=8) and grey literature (n=10) published in English from 1 January 2004 to 31 July 2012. English was found to be the main language of these studies done in this area, and limited translation resources did not allow us to include any non-English publications. The dates chosen reflected the eight years prior to the study in order to reflect a recent picture of the maternal health situation in Myanmar (very few studies exist to further limit the time span and still retain a variety of information sources). Studies on refugees on the Thai-Myanmar border areas were excluded in order to prevent differing service levels between regions that would affect the results. An initial screening was carried out by examining titles and abstracts for relevance. Searching by key words occasionally generated papers with entirely unrelated and different contents, and these were excluded from the review. A second-stage screening was completed on full-length publications which met the above criteria. Document selection was conducted by the primary author in consultation with the tertiary author.

### Data extraction

Data from qualitative and quantitative studies and grey literature were extracted thematically via a line-by-line coding process (23) and entered in a spreadsheet (Table 1). Study designs and outcomes from quantitative studies were also extracted.

### Data analysis

Qualitative data were coded line-by-line, and then categorized into descriptive themes (23). The themes were then regrouped into four further analytical themes based on principal aim and objectives of this study—a process based on the work of Thomas and Harden (24). These four themes were underlying factors of MMR in the eastern regions relating to: the health system, conflict, politics, and socioeconomics. The quantitative data were coded into the descriptive themes described above and translated into the analytical themes to seek sources of convergence (25). Quantitative data were presented in a narrative style as well as numerically, to address the objective of each study. Statistical meta-analysis was beyond the scope of this study, and there were only a few quantitative studies to meet the requirements for such an analysis. Findings from the quantitative studies helped complement the themes emerging from the qualitative studies.

### Data synthesis

Analytical themes were generated based upon the combined qualitative and quantitative findings to synthesize the findings into an overarching framework (23). Some themes were deductively created (e.g. political factors) where the authors postulated broader themes likely to be present in the literature while others evolved through an inductive process where specific findings in the literature were generalized into a broader theme (e.g. socioeconomic factors).

**Table 2.** Study summary

Author, year and title	Design and sampling	Factors measured	Outcome
Back Pack Health Worker Team (BPHWT), 2010 Diagnosis: Critical health and human rights in eastern Burma (4)	Survey design Sample: mothers of youngest child interviewed Sampling frame: 325,094 people and 57,950 households from 273 randomly-selected villages from 4 states and 2 divisions—between Sept 2008 and Jan 2009	Anthropometry Malaria/diarrhoea Landmine injuries Reproductive history	MMR 721 deaths/100 000 livebirths Pregnancy and childbirth=2.2% of all deaths Malaria=24.7% of deaths Diarrhoea=14.9% of deaths Landmines and gunshot=2.3% 14.7% met criteria for iron supplementation 78.2% of women didn't use modern contraception 18% malnourished 1/3 of households experienced at least 1 HRV in last year Forced labour in household=2.5X higher chance of infant death
BPHWT, 2006 Chronic emergency: health and human rights in eastern Burma (8)	Survey design and semi-structured interviews Sample: household heads Sampling frame: 140,000 people and 2,000 households taken from 100 clusters	Anthropometry Malaria/diarrhoea Contraceptives Iron supplements Mortality rates Forced labour Attacks Theft/destruction of livestock Denial of care Forced relocation	MMR 1,000-1,200 deaths/100,000 livebirths Lifetime risk of maternal death: 1 in 12 12.6% positive for malaria 9.8% suffered diarrhoea 4% of IDP women had access to EOC Approx 80% never used contraceptives 40% received iron supplementation Crude birth rate 41.8/1,000 % of households who experienced: forced labour 32.9% soldier violence 1.9% forced displacement 9% food destroyed/looted 25.7% households landmine injuries/deaths 0.3%
Mullany *et al.* 2007 Population-based survey methods to quantify associations between human rights violations and health outcomes among internally-displaced persons in eastern Burma (30)	Survey design Sample: household heads Sampling frame: 129,000 people and 2,000 households taken from 100 clusters	Anthropometry Malaria/diarrhoea Mortality rates Forced labour Theft/destruction of food Denial of care landmine injury Forced relocation	Almost 1/3 households reported forced labour 8.9% forced displacement 25.2% theft/destruction of food Multiple HRVs in 14.4% of households Forced displacement= 2.8 X increased risk of child mortality 3.22 X increased risk of malnutrition 3.89 X increased risk of landmine injury Theft/destruction food supply= 1.58 X increase in crude mortality 1.82 X increased risk of malaria parasitemia 1.94 X increased risk of child malnutrition 4.55 X increased risk of landmine injury
Mullany *et al.* 2008 Access to essential maternal health interventions and human rights violations among vulnerable communities in eastern Burma (13)	Survey design Sample: 3,000 women between 15 and 45 years Sampling frame: 60,000 people from 12 project communities	Access to antenatal and postnatal care, family planning services Skilled attendance at birth Anthropometry Malaria/Hb Forced labour Forced relocation	88% home delivery 5.1% skilled delivery 39.3% any antenatal visits, 16.7% at least 4 visits 21.6% bednets 11.8% receipt of iron supplements >60% unmet need 7.2% Pf malaria >60% Hb less or equal 11g/dL >50% anaemic (women) % of households experiencing: forced labour 32.1% forced displacement 10% Older women reported 6.9 pregnancies Mean MUAC (women) 24.4 cm, <22.5 cm in 19.3% Anaemia: 1.51 times higher (95% CI 0.95-2.40) among women reporting forced displacement 7.47 times higher (95% CI 2.21-25.3) among women reporting food security violations Odds of receiving no antenatal care 5.94 times higher (95% confidence interval [CI] 2.23-15.8) among forcibly displaced women
Mullany *et al.* 2010 Impact of community-based maternal health workers on coverage of essential maternal health interventions among internally-displaced communities in eastern Burma: The MOM Project (28)	Survey design (before and after) Sample: women between 15 and 45 years 2,889 in 2006, 2,442 in 2008 Sampling frame: 60,000 people from 12 project communities	Demographics Access to antenatal and postnatal care, family planning services Skilled attendance at birth Anthropometry Malaria/Hb Forced labour Forced relocation	Similar demographics between 2 groups Following the intervention: More likely to receive antenatal care (71.8 vs 39.3%); postnatal care (PRR)=1.83 (95% CI 1.64-2.04) Double rate of postnatal care (PRR)=2.07 (95% CI 1.81-2.37) Unmet contraceptive need down to 40.5 from 61.7% PRR=0.65 (95% CI 28%-40%) Modern contraception increased from 23.9% to 45.0% PRR=1.88 (95% CI 1.63-2.17) Attendance at birth increased almost 10 X from 5.1 to 48.7% PRR=9.55 (95% CI 7.21-12.64) More likely to undergo malaria screening (55.9% versus 21.9% PRR=2.88 (95% CI 2.15-3.85)
Grundy *et al.* 2012 The responsibility to protect: inequities in international aid-flows to Myanmar and the Democratic People's Republic of Korea and their impact on maternal and child health (17)	Review study Sample: public health and health systems literature OECD and WHO databases Primary data via field visits to North Korea and Myanmar	Regional trends in overseas aid-flows Maternal and child health indicators National health system funding	Despite higher child and maternal mortality rates, aid-flows up until 2008 were significantly lower in Myanmar than other SE Asian countries Aid-flow per capita to Myanmar among the lowest in the SE Asian region (OECD 2009) Myanmar largely excluded from multi-lateral aid Health system on the verge of collapse due to neglect by national government and international community
Teela *et al.* 2009 Community-based delivery of maternal care in conflict-affected areas of eastern Burma: perspectives from lay maternal health workers (29)	Qualitative design Sample: maternal health workers (MHWs) Focus groups Semi-structured interviews Case studies Informal discussions Questionnaire Unstructured interviews	Qualitative information regarding experience: introduction to and relationships with community, collaboration among health workers, intervention-related topics, coverage and access issues, supply, and logistics problems	Trust necessary for timely care to be achieved Some EOC services can be delivered in the community Challenges to MHC service delivery were overcome (e.g. security problems, transportation issues) Successes: impartial care and consistent support of communities in turmoil, increased MHW confidence, expansion of family planning services, increased collaboration between 3 tiers (Traditional birth attendant, health worker, maternal health worker), expansion of TBA roles in some communities (e.g. prophylactic misoprostol)
Saha 2011 Working through ambiguity: international NGOs in Myanmar (26)	Qualitative design Sample: 15 interviews with INGO, NGO and bilateral donor country personnel and US-based INGO staff, scholars and analysts, and research and analysis	The landscape and arrangements under which organizations operate Whether and how interventions are reaching and impacting those in need Ways human-itarian access can be expanded Ways independence and impact can be safeguarded Examples of effective work appearing Identify/share innovative way to work Explore collaboration	Key challenges uncovered: human rights violations, ongoing conflict, poor public health, lack of governmental will and capacity to provide health services, lack of expatriate staff mobility, fluctuating visa approvals, limited humanitarian space, uncertain restriction status, short-term donor funding Key findings: recent shift in government tone regarding NGOs, INGOs confront serious ethical/operational dilemmas, constitutional and political structural change is promising, humanitarian space has opened significantly since Cyclone Nargis, INGOs and analysts believe in aid delivery without bolstering Government, INGOs believe they're having a positive impact (e.g. building local capacity), donor assistance could be delivered effectively in most parts of Myanmar, there is no optimal way in which to work, capacity-building and participatory development is vital, advocacy to Government is vital, careful use of safeguards important

MMR=Maternal mortality ratio; IDP=Internally-displaced person; EOC=Emergency obstetric care; HRV=Human rights violation; Hb=Haemoglobin; Pf=*Plasmodium falciparum*; MUAC=Mid-upper arm-circumference; OECD=Organisation for Economic Cooperation and Development; WHO=World Health Organization; SE=Southeast; MHC=Maternal healthcare; TBA=Traditional birth attendant; NGOs=Non-governmental organizations; INGOs=International non-governmental organizations; US=United States (of America)

## RESULTS

Table 2 provides a summary of the quantitative (n=5) and qualitative studies (n=2) and review articles (n=1) found through a detailed literature search. Table 3 lists the 11 themes, which were synthesized from these studies (n=8) in combination with a qualitative summary study (n=1) and publications sourced via the grey literature search (n=10). Underlying factors that influenced high MMR in eastern Myanmar could be broadly categorized into the following themes:

### Health system-related underlying factors for MMR in the eastern regions

Access to and availability of maternal healthcare (MHC) services was an underlying factor identified in the review. It appeared in 11 of the 19 publications studied. Grundy *et al*. found that, despite a maternal mortality rate higher than in other southeast Asian countries, per-capita aid-flows to Myanmar were among the lowest in the region (17). They described the health system as being on the verge of collapse in 2012. A report by the Hauser Center suggested that short-term donor funding prevented adequate healthcare service delivery by the participating NGOs in the study (26). For instance, 88% of women delivered their babies at home (13).

In a 2008 study, the authors stated that it was necessary to decentralize the provision of healthcare to properly serve the eastern regions of Myanmar where a community-based delivery system could allow many of the necessary pregnancy and delivery services to be provided (27). The three-tier health worker system used by certain NGOs in the area was designed whereby maternal health workers provided a wide range of MHC services, and health workers and traditional birth attendants (TBAs) provided a narrower range of services (27). This three-tier system has improved the accessibility of care for many women in the region (28). However, the overall lack of availability and of user-friendliness of MHC services was compounded by difficult access (transport, fees, etc.) (13,28).

The presence of MHC services (family planning, antenatal care, emergency obstetric care, postnatal care, postabortion care) was considered to be lacking or problematic in six of the 19 publications. For instance, the BPHWT found that only 4% of respondents had access to emergency obstetric care (EOC) (8) while another study found that only 5.1% had access to skilled delivery (13). Only 16.7% of women surveyed had made the standard minimum of four antenatal care visits in their last pregnancy (13). The odds of receiving no antenatal care were 5.94 times (95% CI 2.23-15.8) more likely among displaced women (13). In a later study, Mullany and colleagues found that, after implementing a community-based MHC project, women were more likely to receive antenatal care (71.8% vs 39.2%), were twice as likely to receive postnatal care, and were 10 times more likely to have skilled attendance at birth (28). In a qualitative study based upon the same project (29), the authors found that some EOC services can be delivered in a community setting, particularly with increased collaboration between the three tiers of service providers and an expansion of the role of TBAs in certain regions.

Contraception was identified as an area of concern by four of the publications. Approximately 80% of women in two BPHWT studies had not used contraception (4,8). A study by Mullany *et al.* found that greater than 60% of women had an unmet need for contraception (13). In the eastern regions, older women reported an average of 6.9 pregnancies, with 88% of their most recent births occurring at home (13). Forced relocation was associated with 6.1 times decreased use of contraception (8). The MOM project implemented community-based MHC, which resulted in a decrease in unmet contraceptive need from 61.7% to 40.5% of the women surveyed, and an increased use in modern contraception from 23.9% to 45% (28).

**Table 3.** Extracted themes: influences on maternal mortality in eastern Myanmar

Influences on maternal mortality in eastern Myanmar	
Analytical themes	Descriptive themes
Conflict-related underlying factors of MMR in the eastern regions	Personal violation or violence (4,8,13,22,26,27,29,30,35,36,39,44-47)
	Removal of food and the means to purchase food (4,8,13,22,26-30,35,36,39,44,46,47)
	Forced labour (4,8,13,22,26-30,35,36,39,44,46,47)
	Forced relocation or displacement (4,8,13,22,26-30,35,36,44,46,47)
	Targeting of medical staff, buildings, and supplies (8,13,26,29,44-46)
Health system-related underlying factors of MMR in the eastern regions	Access to and availability of MHC* services (4,8,10,13,17,27-30,35,46)
	Presence of MHC services (4,8,13,17,27,28)
	Contraception (4,8,13,28)
Underlying political factors of MMR in the eastern regions	Limitations placed upon NGOs (4,8,13,21,26,27,29,44-47)
	The lack of health-related data (4,8,26,35,46)
Socioeconomic underlying factors of MMR in the eastern regions	Co-existing morbidity among pregnant women (4,8,13,27,28)

*MHC=Maternal healthcare

### Conflict-related underlying factors for MMR in the eastern regions

Conflict-related factors are difficult to link directly to maternal mortality. However, these factors play an important role in increasing the risk of illness and death in pregnant women in eastern Myanmar. Multiple types of human rights violations (HRVs), such as forced labour, soldier violence, theft or destruction of food supplies, injuries from landmines, and forced displacement were reported in 14.4% of households surveyed by Mullany *et al*. (30). Households experiencing HRVs were also more vulnerable to malaria and anaemia. Deaths as a result of malaria caused by *Plasmodium falciparum* were 2.34 times (95% CI 1.27-4.32) more likely in families having experienced two or more HRVs in the previous year (8). The instability caused by HRVs was found by the Back Pack Health Worker Team (2006) to make it difficult for all individuals, including pregnant women, to prioritize their health, and this was a further barrier to accessing healthcare (8).

Personal violation or violence was identified as a concern in the majority (15/19) of the publications considered. Experience of serious forms of violence was widespread; 0.6% of households in the eastern region reported experiencing a gunshot injury, and another 1.7% experienced a landmine injury in the year preceding one study (4). In an earlier study by the same group, 1.9% of households studied reported experiencing physical assault or violence perpetrated by military personnel (8). The odds of experiencing a landmine injury increased (OR 19.79, 95% CI 2.59-151.2) in households having experience of two or more HRVs in the last year (30). Sexual violence committed by the military was an underlying factor reported in 11 of the 19 publications considered in this study.

The removal of food as well as the means to purchase food was also identified in 15 of 19 publications under study, and this contributed to food insecurity. The Back Pack Health Worker Team (BPHWT) reported destroyed or looted food by the military in 25.7% of households studied (8)—very similar to the rate found by Mullany and colleagues (30). Theft or destruction of food by Myanmar's military (the Tatmadaw) was associated with increased crude mortality (OR 1.58, 95% CI 1.09-2.29), landmine injury (OR 4.55, 95% CI 1.23-16.91), and malarial parasitaemia (OR 1.82, 95% CI 1.16-2.89) (30). Additionally, anaemia was 7.47 times (95% CI 2.21-25.3) more likely in women reporting food security violations (13).

Forced labour, such as portering and road construction, was widespread in both males and females as reported in 15 of 19 publications reviewed. Mullany *et al.*'s study found forced labour in almost one-third of the households (30)—a rate also reported in a subsequent study (13). Forced labour in the preceding year occurred in 32.9% of the households surveyed by the BPHWT in their 2006 study (8). Mullany *et al.* noted that forced labour in the surveyed households was linked to an increased likelihood of death (OR 1.29, 95% CI 0.79-2.12), landmine injury (OR 2.62, 95% CI 0.71-9.61), and malaria diagnosis (OR 1.32, 95% CI 0.78-2.23) in a household member (30). The BPHWT found forced labour to be associated with 1.6 times increased likelihood of diarrhoea in household members in the last two weeks. Mullany *et al*. linked this finding to the necessity of drinking contaminated water and eating in unhygienic conditions (8).

Forced relocation or displacement was an underlying factor present in 14 publications. The BPHWT 2006 study reported that 9% of households had experienced forced displacement in the preceding year (8)—similar to 10% reported by another study (13). Forced relocation was associated with 6.1 times decrease in contraception usage and 4.5 times increase in the chance of being injured by a landmine (8). Mullany *et al.* found forced displacement to be associated with 3.22 times (95% CI 1.74-5.97) higher chance of developing malnutrition, 3.89 times (95% CI 1.01-15.0) higher chance of experiencing a landmine injury, 1.61 times (95% CI 0.75-3.42) increased chance of a death in the family, and 1.58 times (95% CI 0.97-2.57) greater chance of being positive for *falciparum* malaria (30). Additionally, anaemia was 1.51 times (95% CI 0.95-2.40) more likely among women reporting forced displacement (13).

Of the 19 publications studied, seven reported the targeting of medical staff, buildings, and supplies by the Tatmadaw. Teela *et al.* reported how NGO health workers in the eastern regions of Myanmar had to overcome significant security challenges posed by the Tatmadaw to provide maternal healthcare (MHC) services (29).

### Underlying political factors of MMR in the eastern regions

Government-imposed limitations on NGOs working in Myanmar and the hindrances the organizations face were underlined as areas of concern in 11 of the 19 reviewed publications. The Hauser Center's publication identified a number of findings, including a lack of expatriate staff mobility, fluctuating visa approvals, and government-imposed travel restrictions (26). These findings are compounded by limited NGOs’ access to regions of ethnic minority groups within Myanmar and serious ethical and operational dilemmas. Identified ethical dilemmas included how to provide appropriate assistance in a humanitarian environment that is tightly controlled by the Government and the military. The creation of parallel structures (through aid-delivery by multiple sources) was found to be another significant operational dilemma facing NGOs in Myanmar (26) according to a report by Saha (2011).

A lack of health-related data in Myanmar and in the eastern regions in particular (including maternal health data) was identified as an underlying politically-driven factor by 5 of the 19 publications. For instance, NGOs have difficulty in gathering data on the impact of their services due to the restricted mobility of their expatriate staff (26). Myanmar's Ministry of Health regularly underestimates national health indicator data (8), and these data are generalized for the eastern regions where, in fact, minimal data are collected. Therefore, the unique health needs in the eastern regions are neglected.

### Underlying socioeconomic factors of MMR in the eastern regions

Co-existing morbidity among pregnant women in eastern Myanmar was identified as an underlying factor in 5 of 19 publications. In the BPHWT study, 7.3% the of the household heads were found to be positive for *falciparum* malaria (4), a figure closely mirrored by another study which found a rate of 7.2% in the women surveyed (13). Bednets were present in 21.6% of households (13). Malaria was the largest cause of death in another study (8).

Eighteen percent of all those surveyed in BPHWT's 2010 study were malnourished (4) while another study noted that many of those surveyed were malnourished with an average mid-upper arm-circumference of 24.4 cm among adult females (13). The same study (13) found that over 50% of women surveyed were anaemic with haemoglobin counts of less than 11 g/dL in greater than 60%. Only 11.8% of women had received iron supplements during their previous pregnancy (13) while, in another study, only 14.7% of women met international recommendations for iron supplementation (4). An earlier study found that 40% had received iron supplementation (8). The same BPHWT study found that greater than 60% rarely or never used a latrine, and greater than 30% rarely or never boiled their water (8). Diarrhoea was a common finding in the studies, with rates of 6.4% in the two weeks prior to the survey (4) and 9.8% in the households surveyed by the BPHWT (8).

Abortion was not reported or documented as a factor of importance to MMR in any of the publications reviewed.

**Figure.**
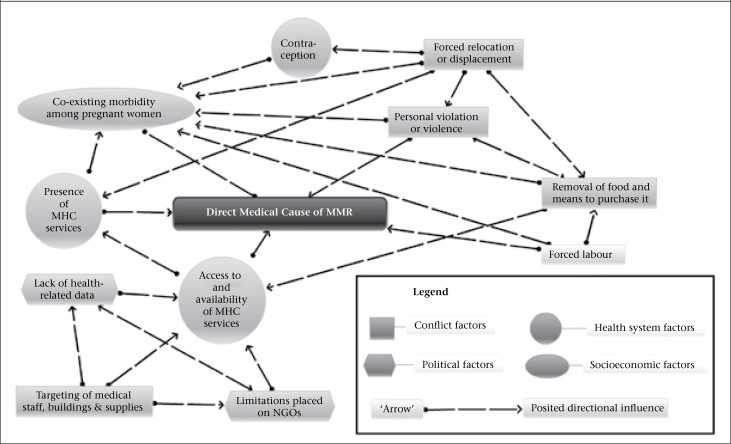
Web of underlying causes of maternal mortality in eastern Myanmar

The studies indicate that the factors that appear to contribute to the higher maternal mortality observed in eastern Myanmar are strongly interconnected.

## DISCUSSION

### Interrelationship of underlying factors influencing MMR

The many inter-relationships among the underlying factors which contribute to maternal mortality in Myanmar's eastern regions are posited in the figure. The model in the figure was created to illustrate the complex inter-relationships of the themes that emerged from the review. The number of inter-relationships is complex, and the authors discuss a selected few in the next section.

### Health system-related underlying factors for MMR in the eastern regions

Access to and availability of MHC services present serious challenges to pregnant women due, in part, to their lack of availability from both Government and NGOs. Travelling to access services increased the likelihood of exposure to soldiers, which risks violence upon their persons, or exposure to landmines. Targeted health workers are also presumably less likely to risk confronting the military in order to ensure the availability of MHC services.

### Conflict-related underlying factors of MMR in the eastern regions

The alarmingly high rates of exposure to HRVs, such as personal violation or violence which includes sexual violence can have a direct impact on the health of pregnant women. For instance, violence perpetrated by soldiers may place a pregnancy in jeopardy. Women who conceive as a result of these encounters may be less likely to access formal maternal healthcare (MHC) services as was the case in Rwanda (31). Even those who did not conceive might still be less likely to access MHC services when they did conceive, due to shame ensuing from their victimization, as suggested in research on victims of rape in northern Uganda (32). Furthermore, anecdotal evidence from Sierra Leone suggested that pregnancies secondary to conflict-related sexual violence may increase abortion-seeking, including unsafe abortions (33).

The removal of food as well as the means to purchase food may directly impact the health of pregnant women in eastern Myanmar since one study found it to be linked with an increased risk of death (30). Furthermore, women foraging for food in the jungle are more likely to come into contact with malaria-carrying mosquitoes (30), soldiers, or landmines. Women injured by soldiers were also less likely to be able to contribute to the raising of crops, which might impact on food security.

Forced labour prevented households from growing their own food or working in a remunerated capacity. Pregnant women forced into such labour were also more likely to be exposed to sexual or physical violence due to their close proximity to soldiers. Forced labour conditions were often unsanitary and unfamiliar, further exposing people to landmines, malaria (30), and other diseases. These conditions also reduced their access to appropriate food sources.

Forced relocation and displacement had a serious impact on food security and nutritional status of pregnant women since households were relocated away from fields and food storage facilities. It also increased women's risk of contracting malaria (e.g. not sleeping under a bednet), a condition which claimed the lives of many displaced pregnant women during the Afghan conflict (34). The odds of exposure to landmines were also increased with forced displacement (8) since women travelled in dangerous areas and foraged foods in unfamiliar territory.

### Underlying political factors of MMR in the eastern regions

The lack of health-related data, particularly in the area of maternal health, from the eastern regions of Myanmar, is closely related to the difficulty that NGOs have in accessing this region. A lack of data on the severity of health concerns affected programme planning (26) and, in turn, the availability of the funding of NGOs. Ongoing conflict made it difficult to conduct research (35) due, in part, to the dangers associated with data collection in the region. A lack of appropriate data also posed challenges for the Myanmar Government and other service-delivery agents to making appropriate decisions regarding which MHC services were most needed. Limited data impacted on planning (26) which contributed to the poorly-tailored and geographically-disparate MHC service provision.

### Emerging political changes

Myanmar has made encouraging changes recently; by-elections won by Aung San Suu Kyi's National League for Democracy and changes to the constitution and political structures suggest a liberalization and democratization of the country. The Government has also softened its attitude towards NGOs by giving them greater access to certain regions within the country (26). Additionally, the Human Rights Commission has been formed, and a number of military members have recently been sentenced for raping and murdering a woman of an ethnic minority group (26,36,37). Finally, a recent drive to produce a national ceasefire with the majority of Myanmar's ethnic armed groups is underway (38). These events all point to the possibility of substantial and real change in Myanmar. However, much remains that is troubling. Human rights violations continue (39), and these crimes remain largely unpunished as civilian courts still do not have jurisdiction over the military (36). The KIA (Kachin Independence Army) alleges that the military is not following the President's peace-promoting decisions in the Kachin region (40). Sporadic fighting persists, and ethnic communities continue to face systemic discrimination from the quasi-civilian state (38,41). Although some reductions in maternal mortality are reported to have been achieved (2,42), many of the underlying factors making MMR high in the eastern regions remain largely unchanged.

### Limitations of the review

The present review was conducted using the available publications that remain limited despite a wide-ranging literature search using a thorough, methodical and replicable process. Publication bias, typical of such reviews (43), was countered through the collection of unpublished documents, such as dissertations and grey literature. The quality of data may also be suspected, based on the data variability from source to source, i.e. Ministry of Health (8). A subset of the publications included in the study focused on the reporting of HRVs in eastern Myanmar where maternal health concerns were a secondary focus or were not directly addressed.

Although the methodological rigour of the studies examined were practical for the conflict-prone areas under consideration, this did not allow us to determine the relative weights of the actual causes of increased maternal mortality in eastern Myanmar. A similar cohort of women receiving services from either the BPHWT or the MOM group (those who conducted the studies in question) could be included in a subsequent study to improve the strength of the results found. It is difficult to make a one-to-one link between some of the conflict-related factors and maternal death from these studies. Nevertheless, these factors can all contribute to an environment that is detrimental to the healthy progress of pregnancy or maternal survival.

### Conclusions

Further progress in Myanmar is essential to reduce maternal mortality in the eastern regions. To reduce the conflict-related underlying causes, measures must be taken to reduce the human rights violations occurring in the eastern regions. The Myanmar Government must develop a comprehensive plan that truly engages the ethnic minority groups in serious dialogue (22), one that can lead to national reconciliation. A call has been put forth by Suu Kyi and other political figures for holding a second Panglong Conference to bring ethnic leaders, together with the Government (39). This could be an important first step.

Furthermore, the Myanmar Government should enact laws which enshrine the rights of pregnant women. Without changing the prevalence of these conflict-related underlying causes (found in the study to be of greatest concern to women's health), the authors suggest that few other measures will have the lasting effect needed to reduce MMR in the eastern regions of Myanmar.

The conflict-related barriers to accessing MHC services are ongoing but can be mitigated. The provision of the government-funded mobile maternal health services, as suggested by Teela *et al.* (29), to the eastern regions, would greatly reduce these barriers. An alternative might be the creation of numerous health “safe zones” where ethnic women could access MHC services free from harassment or molestation by the military. This approach should be complemented by further funding and support from the international community for the existing exemplary cross-border initiatives that currently provide MHC services to this population.

Health system-related underlying factors contributing to high maternal mortality in Myanmar's eastern regions must also be addressed: a greater focus on maternal health provision to the communities in eastern Myanmar is crucial to reducing the unnecessary deaths (13,27). Another essential element of MHC that must be expanded is the provision of family planning services, or, “birth spacing services” in the terms of the Ministry of Health. Contraceptive services could be provided by government-employed community health workers who travel to the villages in which the minority women live. Finally, the underlying socioeconomic factors must not be ignored. Aid and humanitarian agencies must partner with the Myanmar Government, increasing aid-flows desperately needed to combat high maternal mortality in the eastern regions (17). In the process, it will also serve to increase the dialogue between the Myanmar Government and the international community on the urgent matter of addressing maternal mortality in eastern Myanmar.
